# Reply to Comments: A Novel Low-Cost Instrumentation System for Measuring the Water Content and Apparent Electrical Conductivity of Soils, *Sensors*, 15, 25546–25563

**DOI:** 10.3390/s18061742

**Published:** 2018-05-28

**Authors:** Alan Kardek Rêgo Segundo, Marco Jose da Silva, Gustavo Medeiros Freitas, Paulo Marcos de Barros Monteiro, José Helvecio Martins

**Affiliations:** 1 Department of Control and Automation Engineering (DECAT), Escola de Minas, Universidade Federal de Ouro Preto (UFOP), Morro do Cruzeiro, 35400-000 Ouro Preto, MG, Brazil; pmemop@gmail.com; 2 Department of Electrical Engineering (CPGEI), Universidade Tecnológica Federal do Paraná (UTFPR), Av. Sete de Setembro, 3165, 80230-901 Curitiba, PR, Brazil; mdasilva@utfpr.edu.br; 3 Instituto Tecnológico Vale (ITV), Avenida Juscelino Kubitschek, 31, Bauxita, 35400-000 Ouro Preto, MG, Brazil; gustavo.medeiros.freitas@itv.org; 4 Instituto de Educação Tecnológica (IETEC), 30140-138 Belo Horizonte, MG, Brazil; j.helvecio.martins@gmail.com

**Keywords:** dielectric constant, electrical conductivity, auto-balancing bridge, microcontroller, embedded system

## Abstract

In this article we respond to the comments made by Chavanne et al., who have questioned: (i) the name of the technique used; (ii) the ability of the system to determine both soil water content and salinity due to potential instrument biases and choice of sensor frequencies; and (iii) the procedure used to determine temperature effect on readings presented in the article “A Novel Low-Cost Instrumentation System for Measuring the Water Content and Apparent Electrical Conductivity of Soils” (Sensors 2015, 15, 25546–25563). We have carefully analyzed the arguments in the comment, and have concluded that they only partially affect the previous conclusions, as will be discussed in this reply. We show here that the findings and conclusions previously drawn are valid and supported by the many experiments previously conducted.

## 1. Introduction

We thank Chavanne et al. for comments made on our work. This highlights the current need for accurate measurements in the field of porous medium impedance, especially soils. In the following, we respond to each of the points raised.

The instrumentation system for measuring soil parameters such as water content, apparent electrical conductivity, and temperature presented in [1] was developed with the aim of being cost effective, which intrinsically means that no absolute accuracy may be achieved. The main envisioned application is to automate irrigation systems, and consequently, provide farmers with an alternative means of rationing water and electricity use in crops. Thus, the reported system may be seen as the first step towards the widespread use of such technology in field applications in the near future.

In the next section, there is a discussion about the different names found in the literature regarding the measurement technique used in [1]. In Section 3, we give more details about the frequencies and the electronic circuit used in [1]. Section 4 seeks to clarify the issues raised by Chavanne et al. regarding the procedure for assessing the effect of temperature on electrical conductivity measurements. In Section 5, we point out the main conclusions of this work.

## 2. Measurement Technique

Chavanne et al. questioned the name we used for the measurement technique presented in [1]. They assert that a bridge is a zeroing method that provides voltages which are directly proportional to the permittivity (*ε*) and conductivity (*σ*) of the soil.

In [1], we shortly reviewed the methods for measuring impedance at low frequencies (up to the MHz band), and classified them into three categories: current-voltage (or I-V), bridge, or resonant methods. The I-V method is very simple, albeit with low accuracy. The classical bridge method has high accuracy, but due to the need for balance, it is not suitable for fast and continuous measurements. More recently, Chavanne et al. [2] were able to circumvent these limitations. The resonance method has good accuracy, but similar to the bridge method, it needs tuning, and is not suitable for fast measurements. It is noteworthy that all three methods are sensitive to parasitic capacitances (generated by connecting cables, for instance), the elimination of which may require complex circuits.

**Figure 1:**
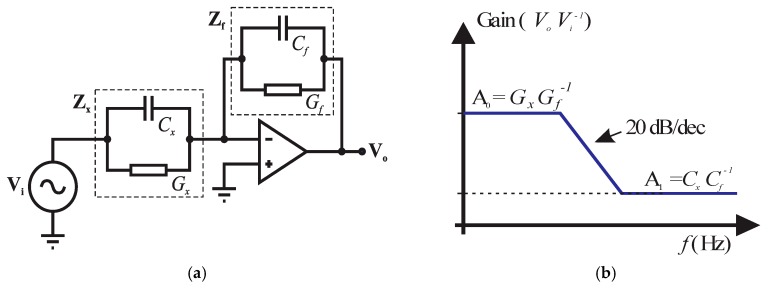
(**a**) Auto-balancing bridge circuit; (**b**) frequency response.

The I-V method consists of applying an alternating voltage **V** to an unknown impedance **Z_x_** in series with a known electric resistance *R*. Thus, **Z_x_** can be determined by measuring the source current **I** and the impedance voltage **V_x_**, and then applying Ohm’s law; or in more practical terms, by measuring the voltages over *R* and **Z_x_**. Traditionally this technique does not need to apply any amplifier circuit, and does not analyze the response at different frequencies to measure the relative dielectric constant (*ε*) and conductivity (*σ*) of the material under test. In [1], we used a variation of the I-V method to obtain higher precision and faster response, while keeping circuit as simple as possible. Moreover, it has a high signal to noise ratio and stray capacitance immunity, capable of measuring small impedances between the electrodes even in the presence of large stray capacitances to ground. The circuit presents these advantages due to the use of a transimpedance amplifier, and in [1], the sum of the currents in the virtual ground of the amplifier is considered to be zero. In addition, the method in [1] performs a comparison between the unknown impedance **Z_x_** and a reference impedance **Z_f_**; hence, it determines *σ* and *ε* by measuring the gain at two different frequencies, each in a distinct plateau (Figure 1). If the unknown and feedback impedances are equal, for example, the gain at both low- and high-frequency plateaus is equal to 1. As the unknown impedance varies with respect to the reference impedance, the gains are changed, and consequently, *σ* and *ε* are determined. Figure 1a,b shows the circuit configuration and its asymptotic frequency response, respectively.

In [1], we state that such a measurement topology has received several names, i.e., synonymously: auto-balancing bridge, self-balancing bridge, transimpedance amplifier converter, or current-voltage converter. Chavanne et al. brought a further designation for that circuit topology, calling it (Modern) Ohm’s Law.

Several papers in the literature referred to the term *auto-balancing bridge* and applied the same circuit topology of Figure 1a, as in [3,4,5,6,7,8,9,10,11,12,13]. Some of them, however, present only a resistance in the feedback, which makes the use of the technique presented in [1] unfeasible, since the frequency response of the circuit does not present two distinct plateaus. Besides, in most cases, commercial equipment (signal generators and oscilloscopes) was used to generate the waveforms and to perform voltage, phase, or current measurements.

In other works, *auto-balancing bridge* refers to standalone equipment such as LCR meters and impedance analyzers, as in [14,15,16]. The principle of operation of the technique is explained, in simplified form, by the same circuit shown in Figure 1a. However, the circuit has greater complexity, and it generally has null voltage and phase detectors, an integrator, and a vector modulator. In addition, other authors use the term *auto-balanced bridge* only when referring to commercial impedance measurement equipment, without giving details about the circuit, as in [17,18,19,20].

The articles [16,21,22,23,24] present the development of a device whose technique is called *digital auto-balancing bridge circuit*. The design uses FPGA both to excite and to realize signal measurements. An algorithm controls the output voltage based on the amplitude and phase difference with respect to the input signal. The digital signal-processing algorithm replaces the analog circuits such as null voltage and phase detectors, integrator, and vector modulator of the analogue auto-balancing bridge circuit.

Several works [25,26,27,28,29,30,31] also use the term *self-balancing bridge* concerning impedance measurement circuits. Besides, there are several different types of circuit topologies in these works.

In addition, the name *charge amplifier* is used in [32,33], where the minimization of the parasitic capacitances effect is discussed in more detail.

We fully agree with Chavanne et al. that the two techniques presented in Section 2.2.3 of their comment are different (see Figure 5 of the comment). However, there are advances in the way that the circuit was used in [1], compared to the technique described by Chavanne et al. as (*Modern*) *Ohm’s Law*. In [1], we evaluate the gain of the circuit at two different frequencies, in order to perform the measurements based on the frequency response of the circuit. In [34], Chavanne et al. have used only one resistor in the feedback of the operational amplifier, not a capacitor in parallel with a resistor, which better represents the soil, and more importantly, establishes the frequency response of the circuit with two plateaus, which allows the use of the technique presented in [1]. In addition, we have developed a fully embedded solution through a microcontroller. That is, we do not use a waveform generator, oscilloscope, or computer to carry out the measurements.

Hence, according to the literature review presented above, the circuit topology applied in [1] could have been designated by different terms, but we decided at that time that *self-balancing bridge method* was appropriate. However, as the objective was to develop a simple and low-cost circuit, some simplifications were made, compared to the topologies that use null voltage and phase detectors, an integrator, and a vector modulator. Instead, to determine *ε* and *σ*, the gains (*V*_0_*V_i_*^−1^) of the circuit were measured at each of the two-frequency response plateaus shown in Figure 1b. Also, an 8-bit microcontroller is part of the measurement circuit and serves to excite the system with two different frequencies, controlling the control bits of the multiplexer, performing the analog-digital conversions of the signals, calculating *ε* and *σ* using the calibration models, and providing measurements digitally via the UART. Furthermore, in the system developed in [1], the sensor does not emit voltage signals directly that are proportional to *ε* and *σ*. Instead, the microcontroller already supplies the UART signals to the XBee network formed between the sensors, an irrigation control module, and a computer.

In summary, based on these explanations, it was observed that the circuit presented in [1] has received several names in the past. Also, the circuitry does not have a topology equal to an LCR meter or commercial impedance analyzer. It is a simple and low-cost alternative for automating irrigation systems. We agree with Chavanne et al. that the use of the name *bridge* is confusing, because according to the literature, it is used to describe both the circuit presented in [1] and commercial meters. However, given the results obtained in [1], and the purpose of the project, i.e., to be simple and cost effective, the circuit nomenclature has little relevance, since in the past several names were assigned to the same circuit topology.

## 3. Choice of Sensor Frequencies and Instrument Bias

In [1], the frequencies of 100 kHz and 5 MHz were used to measure *σ* and *ε*, respectively. Chavanne et al. suggest that the frequency used to measure *ε* was very low, which may lead to uncertainties due to many relaxation phenomena.

However, Sen Wang et al. [35] report an experiment on the frequency-dependent properties of soil. According the authors:

Rules of changes in soil resistivity and dielectric constant with current frequency applied at various moisture content levels are identified by measuring the frequency-domain complex impedance of the soil samples, and by numerical fitting to the frequency-dependent properties of the soil electrical parameters. Subsequently, the following conclusions are reached:

(1) The changes are great in soil resistivity and dielectric constant when the frequency is low (below 100 kHz). When the frequency is increased, changes in soil resistivity and dielectric constant with frequency gradually slow down until they become constant.
(2) At the low-frequency band (below 100 kHz), the effect of the moisture content of the soil on the frequency-dependent properties of resistivity and dielectric constant is great, whereas at the high-frequency band, the effect of the moisture content of the soil on the frequency-dependent properties of resistivity and dielectric constant is minimal.

**Figure 2:**
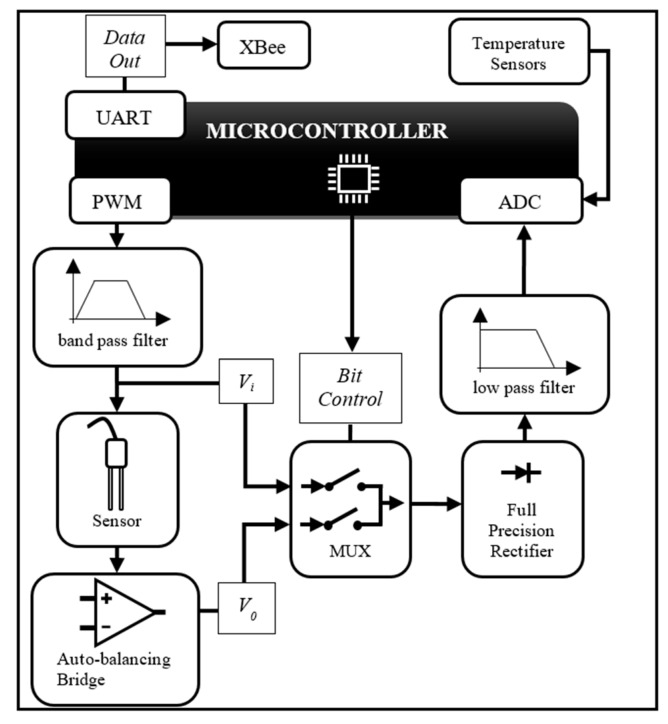
Schematic diagram of the sensor measurement circuit.

The electronic circuit developed in [1] allows for modifying the excitation frequency via the microcontroller firmware, since it comes from a PWM signal. In the current design, a microcontroller with a PWM of maximum frequency of 5 MHz was used. However, by switching the microcontroller and changing its firmware, this frequency can be increased without the need to modify the electronic design, albeit perhaps with the disadvantage of making the new circuit more expensive. Figure 2 shows the diagram of the electronic circuit.

The circuit uses two different frequencies to measure *σ* and *ε*, since instead of measuring the phase of the signal, the system is based on the (much simpler) measurements of voltage gains at each frequency, i.e., 100 kHz for *σ* and 5 MHz for *ε*, respectively.

We agree with Chavanne et al. in that *σ* of the soil depends on the frequency used, and furthermore, the system presented in [1] does not allow direct use of the proposed methodology by Hilhorst [36] to determine the conductivity of the pore water of soil (*σ_p_*), because the measurements of both *σ* and *ε* must be performed at the same frequency.

In [1], we considered the apparent soil electrical conductivity (*σ*) may be defined as the bulk soil conductivity, which is influenced by salinity, saturation percentage, water content, bulk density, organic matter, cation exchange capacity, clay content and mineralogy, and temperature [37]. In [1], it does not show how to perform estimates of *σ_p_* from *σ* measurements, because it was beyond the scope of the work, which consisted only of measuring *σ* and *ε*. This could be undertaken by using empirical relationships, as presented by [37,38], i.e., by using theoretical models and soil characteristics, as in [39], or by direct calibration between *σ* and *σ_p_*, which is more labor-intensive.

Regarding conductivity measurements, the system was actually only evaluated up to 80 mS·m^−1^. In spite of this, the results presented in [1] are not compromised, since the conductivity magnitude order of the used soils was smaller. In a future publication, we can evaluate the behavior of the system at higher frequencies and when subject to higher conductivities, as well as the influence of conductivity on electrical permittivity measurements. Besides that, some precautions should be taken regarding instrument bias, such as the evaluation of the effect of wire and electrode inductances at high frequencies, as well as the electrode impedance at low frequencies, as suggested by Chavanne et al. 

## 4. Temperature Influence

Chavanne et al. question the way in which we evaluated the effect of temperature on the measurements of *σ*. During this procedure, a Del Lab DL-150P was used to determine the conductivity of the NaCl solutions at 25 °C, using the device’s automatic temperature correction, performed with a temperature sensor inserted into the solution. This procedure was performed in the laboratory with air conditioning, at an ambient temperature of approximately 25 °C.

After determining the solution’s conductivity, the low frequency gain (*A*_0_) was correlated with the conductivity of the solutions at an ambient temperature of approximately 25 °C. Therefore, the developed system became capable of performing conductivity measurements only at 25 °C.

Next, to evaluate the temperature effect of the solutions on the conductivity measurements using the developed sensor, a climatized chamber was used to vary the temperature of the sensor element and the solutions (from 5 °C to 45 °C), but not the temperature of the measuring circuit, which was outside the chamber. It is noteworthy that the experiment was performed in a laboratory with air conditioning, and therefore, the measuring circuit remained at a constant temperature of approximately 25 °C.

When evaluating the effect caused by the temperature variation of the solutions, we obtained a correction model. Then, the same procedure was performed with soil samples. Considering the measurement of soil electrical conductivity at 25 °C as a reference, and after applying the correction model found for the solutions in the conductivity measurements of soils (sandy and clay), we observed a significant improvement in the results.

This procedure was completely empirical, and did not intend to provide a single model to explain all temperature effects in soils, because it was used only in two types of soil. It partially minimizes the effect of temperature drift, mainly on the water electrical conductivity in the pores. We agree that modeling the effect of temperature changes on the electrical conductivity of soil bulk is complex, because it must take into account other effects, such as the amount of water and dissipative effects, as well as the effect of the transport of ions by water retained in the pores.

Chavanne et al. [2] reported that the model to correct the temperature found in [1] presents the same temperature drift as presented by commercial instruments, as used for measuring electrical conductivity of solutions.

As reported by Chavanne et al., during field tests, variations of *ε* with temperature over the diurnal cycle were also observed. In a recent publication [40] we empirically minimized this effect on the volumetric soil water content (*θ*) using multiple linear regression, including the temperature of the measuring circuit (*T*) in the model to estimate the volumetric soil water content at 20 °C (*θ*_20°C_), according to:

(1) $$\theta_{20{}^{\circ}C}=2.84624-0.15635T+1.02103\theta$$

**Figure 3:**
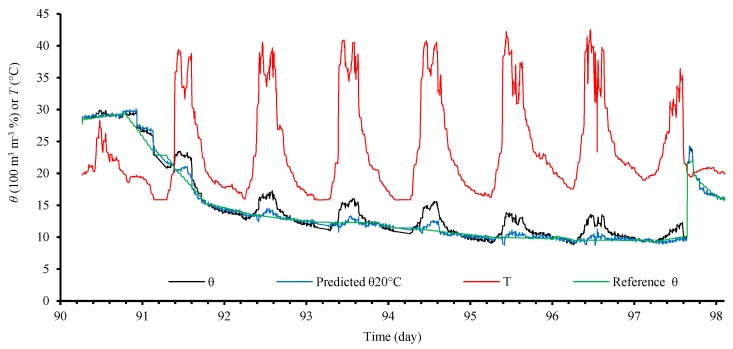
Observed *θ*, predicted *θ*_20°C_, reference *θ* and *T* over time.

It is noteworthy that the measurement circuit has a LM35 sensor in its circuit board, which is installed about one meter above the ground. The results are illustrated in Figure 3.

The tests were carried out in 2016. The measurements were sent through the XBee network every two minutes to a computer, which, in addition to storing the data, makes them available on the Internet (www.irrigosystem.ufop.br). Since the current (energy consumption) of the system is low (in the order few microamperes in sleep mode, and about 100 mA in operation, including the XBee PRO), it is suitable to be powered by a battery.

The soil temperature sensor was not used in [40], because in [1], a relation was obtained to minimize the effect of the temperature only on *σ*, and not on *ε*. Therefore, in [40], only the effect of the temperature of the electronic circuit on soil water content measurements was evaluated. Although the effect of the diurnal cycle on *σ* has not been addressed in [40], soil and electronic circuit temperatures should be taken into account in this case.

## 5. Conclusions

In this paper we present a response to the comment made by Chavanne et al. in relation to the paper [1].

Concerning the technique name used in [1], it was verified that there are several names and variations for the circuit in the literature; this is a direct consequence of the particularities of the sensor element, the application, or the applied frequencies. However, all types of circuits and systems presented in Section 2 are based on the same operational amplifier topology, which makes use of the virtual ground (and sometimes associated circuits) to enhance performance (in terms of frequency range or accuracy) of the simple I-V method. Hence, based on the measurement of amplitudes, phase, or current, the impedance of the material under test is determined. As the results presented by [1] have shown the proposed technology to be an efficient alternative to automate irrigation systems, especially due to the circuit simplicity, the satisfactory accuracy, and the associated low-cost, the authors understand that the discussion of the name of the technique does not diminish the work’s relevance.

The frequencies chosen were satisfactory for the evaluated soils, as well as for fulfilling the main function of the system, which is to automate irrigation systems. However, because the circuit can only be upgraded by changing the microcontroller, tests with frequencies greater than 5 MHz, as well as the influence of conductivity on electrical permittivity measurements, can be envisioned in future investigations. Besides that, some precautions should be taken regarding instrument bias, such as the evaluation of the effect of electrodes and wire impedances.

In [1], no method is provided to figure out the conductivity of the pore water of soil (*σ_p_*) through the measurement of the electrical conductivity (*σ*) in the soil bulk. Such a measurement could be made empirically or theoretically, based on soil characteristics, or even by a direct calibration.

Finally, during the procedure for temperature effect correction, the instrumentation circuit remained outside the climatized chamber at a constant temperature of approximately 25 °C; only the temperature of the sensor element and the solutions were changed. Therefore, the temperature effect on the measuring circuit was not evaluated in [1], but only on the sensor element inserted in the solutions. In addition, the results showed that after applying the correction model (found empirically for NaCl solutions) in the soil samples, the results improved because the root mean square error decreased significantly. However, this is not a model that is able to explain all temperature effects on soils. It is simply an empirical relationship, based on temperature drift of the solutions.
